# Prospective associations between child screen time and parenting stress and later inattention symptoms in preschoolers during the COVID-19 pandemic

**DOI:** 10.3389/fpsyg.2023.1053146

**Published:** 2023-05-25

**Authors:** Maíra Lopes Almeida, Gabrielle Garon-Carrier, Eda Cinar, Giana Bitencourt Frizzo, Caroline Fitzpatrick

**Affiliations:** ^1^Département de l’enseignement au préscolaire et au primaire, Faculté d`Éducation, Université de Sherbrooke, Sherbrooke, QC, Canada; ^2^Département de psychoéducation, Faculté d`Éducation, Université de Sherbrooke, Sherbrook, QC, Canada; ^3^Departamento de Psicologia do Desenvolvimento e Personalidade, Instituto de Psicologia, Universidade Federal do Rio Grande do Sul, Porto Alegre, Brazil; ^4^Department of Childhood Education, Faculty of Education, University of Johannesburg, Johannesburg, South Africa

**Keywords:** screen time, digital media, inattention, early childhood, pandemic (COVID-19)

## Abstract

**Introduction:**

Child attention skills are critical for supporting self-regulation abilities, especially during the first years of life. On the other hand, inattention symptoms in preschoolers have been associated with poor school readiness, literacy skills and academic achievement. Previous research has linked excessive screen time with increased inattention symptoms in early childhood. However, most research has only focused on TV exposure and did not investigate this association during the COVID-19 pandemic. This atypical context has increased screen time in children worldwide, including preschoolers. We hypothesize that higher levels of child screen media and parenting stress at age 3.5 will be associated with higher child inattention symptoms at age 4.5.

**Method:**

This study draws on participants followed longitudinally over the span of 2-years for an investigation of Canadian preschoolers’ screen media use during the pandemic (*N* = 315, 2020). A follow-up with this sample was completed in 2021 (*N* = 264).

**Results:**

Analyses using multiple linear regression, revealed a positive association between child screen time at age 3.5 and inattention symptoms at 4.5 years. Parental stress was also positively associated with child inattention symptoms. Associations were observed above individual (child age, inhibitory control, and sex) and family (parent education and family income) characteristics.

**Discussion:**

These results confirmed our hypothesis and highlight that preschooler screen use and parenting stress may undermine attentional skills. Since attention is a crucial component for children development, behavior and academic outcomes, our study reinforces the importance for parents of adopting healthy media habits.

## Introduction

Child attention skills evolve dramatically during the first 5 years of life (Diamond, 2002) and are critical for school outcomes from preschool through university, including job success (Diamond, 2014). During the preschool years, children progressively learn how to sustain their attention while engaging with their social and physical environment (Shannon et al., 2021). As attention skills evolve, children become more alert and sensitive to external stimulus. This alertness helps them identify task-relevant stimuli, and purposefully engage or disengage with a task (Corkin et al., 2021). Attention skills are also foundational for children’s burgeoning awareness of the world and their ability to regulate their thoughts and feelings (Posner and Rothbart, 2007). Thus, the development of attentional skills in early childhood has been directly linked to improved effortful control, working memory, and emotion regulation which can then underlie behavioral and cognitive outcomes later in life (Nigg, 2017).

### Child inattention and preschooler outcomes

Attention deficit hyperactivity disorder (ADHD) is one of the most common neurodevelopmental disorders during childhood (Cerrillo-Urbina et al., 2018). Based on epidemiological data, the prevalence of inattention symptoms in preschoolers ranges from 1.3 to 3.9% (Spira and Fischel, 2005; Alhraiwil et al., 2015). In general, symptoms of inattention are related to failing to pay close attention to details, trouble in organizing tasks, careless mistakes in activities, and being easily distracted (American Psychiatry Association, 2013). Hence, preschoolers who have poor attention are most likely to experience a lower level of school readiness (Perrin et al., 2019), low literacy skills (Sims and Lonigan, 2013; Hume et al., 2016), and academic achievement (Spira and Fischel, 2005; Duncan et al., 2007). Even subclinical levels of inattention result in a decline in academic attainment (Sasser et al., 2015). Being inattentive has been negatively correlated with child language comprehension (Parks et al., 2021) and uniquely contributes to lower levels of social competence above and beyond the related constructs of hyperactivity and impulsivity (Parks et al., 2021). Previous studies have also linked inattention to lower emotion regulation, lower executive functions, higher rates of oppositional-aggressive behaviors in preschoolers, worse eating habits, and worse general health, which can compromise developmental and health outcomes throughout childhood (Diamond and Lee, 2011; Ebenegger et al., 2012; O’Neill et al., 2017; Landis et al., 2021).

### Preschooler screen time and inattention

Previous studies have identified several lifestyle factors that may influence the development of child attention. In particular, studies have found that greater preschooler TV viewing contributes to increases in inattention symptoms (Cao et al., 2018; Mostafa, 2019; Li et al., 2020). Research has mostly focused on TV viewing rather than newer media (Christakis et al., 2018). However, the adverse effect of portable devices, such as smartphones and tablets, on attention has also been documented (Konok et al., 2021). According to other studies, preschoolers exposure to screens can lead to increases in inattention symptoms (Tamana et al., 2019; Hetherington et al., 2020; Xie et al., 2020; McArthur et al., 2021a). When investigated over a 22 month periods, multiple exposure to media, such as background use and screen time, significantly decreased child focused attention (Gueron-Sela and Gordon-Hacker, 2020). It is important to note that the data analysed in the aforementioned studies was collected before the pandemic. Child screen time of all ages significantly increased during the COVID-19 pandemic (Bergmann et al., 2022). Globally, an increase from 0.75 h to 6.5 h a day has been reported (Toombs et al., 2022). In Canada, this increase ranged from 2.6 h to 5.9 h a day (Toombs et al., 2022).

### Family and child characteristics, preschooler screen time, and inattention

Screen time and inattention are related to child and family characteristics. For example, media use can vary based on age and sex, with boys and older children being more likely to have longer screen time (Duch et al., 2013; Atkin et al., 2014). Children presenting more difficult temperaments are also exposed to more screen time (Corkin et al., 2021). Similarly, inattention can be affected by child individual traits (e.g., cognitive capacity and children neurodevelopment) (Sasser et al., 2015; O’Neill et al., 2017). Child deficits in inhibitory control have also been linked to increased risk of developing inattention problems (Barkley, 1997). Indeed, poor inhibitory control in preschoolers was predictive of later attentional disorders (Miller et al., 2019). The larger social context of families is also likely to contribute to child screen time and their development of symptoms of inattention. For instance, children from lower income families spend more time using media (Ribner et al., 2017). Low parental education and low family income are also negatively associated with child attention regulation (Mistry et al., 2010).

Contextual factors, such as the COVID-19 pandemic and the parent stress experienced during this atypical historical event are also likely to have contributed to child screen use and inattention. Lockdown measures during the COVID-19 pandemic have resulted in numerous disruptions to family life and have increased parenting stress and family screen use (Hartshorne et al., 2021). Before the pandemic, parenting stress was already associated with greater child screen use (Shin et al., 2021) and less likelihood parental limit and rule setting on the quantity of media intake by children (Walton et al., 2014). Along the same lines, family stress can influence the development of attention in the preschool years (Greenhill et al., 2008). Data collected with 878 parents in the pandemic context showed that parent distress significantly predicted children’s inattention during this period (Marchetti et al., 2020).

### The current study

Previous studies have shown that greater screen time intake by preschoolers can comprise their development of attention skills. However studies have focussed mostly on television exposure, have not considered the role of parenting stress, and have been conducted pre-pandemic. Since inattention has been linked to several adverse developmental and academic outcomes throughout childhood and screen time has increased considerably during the pandemic context, it is crucial to better understand this association while controlling for characteristics of the child and family environment. The present study therefore aims to examine the contribution of preschooler screen time and parenting stress during the COVID-19 pandemic to later inattention symptoms. Individual child (e.g., age, inhibitory control, and sex) and family (e.g., parents’ education, family income) characteristics are included as covariates.

## Method

### Sample

In the context of a larger longitudinal study, parents with children between the ages of 2 and 5 (mean age 3.46) completed an assessment of child digital media use in the spring of 2020 during the first wave of the COVID-19 pandemic (2020, *N* = 315). Participants were recruited by distributing eye-catching posters and flyers to preschools and pre-kindergarten classes, through sign-up sheets and presentations given at preschool and pre-kindergarten registration nights, a Facebook page, and newspaper and radio advertisements. Data was collected during a provincially declared state of emergency and lockdown in the province of [omitted for non-identification of authors], Canada. Mothers were the primary respondent in most cases (*N* = 295 or 93.4%). Most respondents reported being married (82%), born in Canada (91%), and White (90.5%). Our sample contained slightly more boys (*N* = 170) than girls (*N* = 145). Finally, our sample was predominantly English-speaking, with 88.1% (*N* = 280) reporting that English is the main language spoken in their home. A follow-up was carried out 1 year later in 2021. The outcome variable data (*n* = 264, 2021, 83% retention rate) were collected 1 year later (mean age of 4.5 years).

### Procedure

Parents completed the Media Assessment Questionnaire (MAQ, Barr et al., 2020) when children were 3.5 and 4.5. This is a web-based assessment of family media exposure that includes questions on child and family characteristics and child screen use habits. For the purpose of our study questions on child inattention were integrated to our online questionnaire. This assessment has been described in detail elsewhere (Barr et al., 2020). All measures are described in more details below. The present research was approved by two Ethics Committees at [omitted for non-identification of authors] and [omitted for non-identification of authors]. Informed consent to participate was obtained from parents.

### Measures

#### Child inattention

Parents reported inattention symptoms in preschoolers when they were 4.5. More specifically parents reported the extent to which their child had shown inattention symptoms over the last 6 months using the following items: Was unable to concentrate; could not pay attention for long; Was inattentive and; Was easily distracted, and had trouble sticking to any activity. Participants responded using a 3-point Likert scale ranging from Never or not true scored as 1, to Sometimes or somewhat true scored as 2, and Often or very true, scored as 3. Items were derived from the Child Behavior Checklist and Preschool Behavior Questionnaire (Achenbach et al., 1987). We calculated the mean scores and treated this variable as a continuous variable, in which higher scores reflect higher levels of inattention. The Cronbach’s alpha for these inattention items was *α* = 0.74.

#### Child screen time

Parents reported the average amount of time children spent doing each of the following on weekdays and weekend days separately: (1) watching TV or DVDs; (2) using a computer; (3) playing video games on a console; (4); Using an iPad, tablet, LeapPad, iTouch, or similar mobile device (excluding smartphones); or (5) Using a smartphone. Response options included: (1) Never; (2) Less than 30 min; (3) 30 min to 1 h; (4) 1–2 h; (5) 2–3 h; (6) 4–5 h; (7) more than 5 h. We then converted these categorical responses into variables reflecting the number of hours spent with each type of media device. Our approach involved using the midpoint for each response range, except for “Never” where a score of 0 was used, and “5 or more hours a day” where a more conservative score of 5 was used. Weighted daily averages of time spent with each type of media device were then created by multiplying weekday estimates by 5 and weekend day estimates by 2 and dividing the total by 7. Last, we estimated an overall daily screen time by summing the weighted daily average across media devices.

#### Parenting stress

Parents also completed the parenting distress subscale of the Parent Stress Index (Abidin, 2012). In total, parents completed 12 items (i.e., I find myself giving up more of my life to meet my child’s needs than I ever expected). Items were rated on a 5-point Likert scale as: 1 (strongly disagree); 2 (disagree); 3 (not sure); 4 (agree); or 5 (strongly agree); and were then summed to create a total score (Cronbach’s alpha = 0.85).

#### Child and family characteristics

Parents reported their child’s sex and age in years. Child sex was categorized as: (0) Boys and (1) Girls. Parents also reported their level of education and family income. All these variables were collected during Time 1. Education reflects the highest school grade completed by the parent. Responses were categorized as either: (1) High school or college vocational; (2) Undergraduate; or (3) Graduate degree. Income was categorized as either (1) less or equal than 59.000 CND; (2) 60,000 or higher CND.

Child temperamental attention was measured at 3.5 years using the Children’s Behavior Questionnaire—Short Form (Putnam and Rothbart, 2006). In the present study, child inhibitory control was measured based on six items (i.e., Can wait before entering into new activities if s/he is asked to). The short version uses a 7-point Likert scale ranging from 1 (extremely untrue of your child) to 7 (extremely true of your child). Cronbach’s alphas were 0.79.

### Data analytic strategy

We use multiple linear regression to estimate associations between child screen time and parenting stress at age 3.5 years and inattention symptoms at 4.5 years, while controlling for child and family confounders. We use continuous measures of screen time to increase our ability to directly compare our effect sizes with those of previous studies (Madigan et al., 2019; Tamana et al., 2019). This analytical strategy has been used by others (Orben and Przybylski, 2019).

## Results

### Attrition analysis

Retained and unretained participants did not significantly differ in screen time, inattention, child sex and age, inhibitory control, parenting stress, and family income. However, parents with a university degree were more likely than those with a high school/vocational degree to remain in our sample at the follow-up, *χ*(1)^2^ = 4.24. The proportion of missing data on the outcome variable was 16.2%. Missing data were examined with the MVA module in SPSS. We computed Little’s test to evaluate if data were missing completely at Random (MCAR). This test was non-significant (*χ*^2^ = 1.75, df = 4, *p* = 0.78) indicating that data could be deemed missing completely at random. To reduce the bias due to the attrition rate and to maintain statistical power, we carried out multiple imputations using SPSS. The results of regression represent pooled estimates over five imputed estimated data sets.

### Sample characteristics

Descriptive statistics and frequencies for all variables are presented in Tables 1, 2.

**Table 1:** The mean and standard deviation for continuous variables in the model.

	Mean (95% CI)	SD	Min-max	*N* (% missing)
**Predictors**				
Children screen time (hours)	3.44 (3.15–3.74)	2.45	0–10.4	315 (0%)
Parenting stress	18.02 (17.35–18.70)	5.52	12.00–0.43.00	315 (0%)
**Outcome**				
Child inattention	1.67 (1.31–0.1.73)	0.50	1.00–3.00	264 (16.2%)
**Covariates**				
*Child*				
Age (years)	3.46 (3.36–3.56)	0.84	2.00–5.42	315 (0%)
Inhibitory control	4.60 (4.49–4.71)	0.97	1.33–7.00	315 (0%)

**Table 2:** Frequency distribution for categorical variables in the model.

	%	*n*	Total *N* (% missing)
**Covariates**			
*Household income* (Canadian dollars)			295 (6.3%)
<60,000/year	15.9	47	
≥60,000/year	84.1	248	
*Parent education*			315 (0%)
High school diploma, college or lower	25.7	81	
University degree	74.3	234	
*Child’s sex*			315 (0%)
Boys	54.0	170	
Girls	46.0	145	

### Multiple linear regression

The results of the multiple regression model are presented in Table 3. Child screen time (hours) at age 3.5 during lockdown contributed positively and significantly to child inattention symptoms one year later (*β* = 0.14; *p* = 0.02). Parenting stress at baseline positively predicted child’s inattention symptoms at follow-up (*β* = 0.24; *p* = 0.01). Finally, higher scores on child inhibitory control at age 3.5 was also associated with less inattention symptoms at age 4.5 (*β* = −0.25; *p* = 0.01).

**Table 3:** Predicting the relationship between child screen time and inattention symptoms in preschoolers.

	Child inattention symptoms (time 2)		
	*β*	*p* value	Adjusted *R*^2^
*Predictors* (time 1)			0.18
Child screen time (hours)	0.14	0.02	
Parenting stress	0.24	0.01	
**Covariates**			
Child’s age (years)	0.11	0.07	
Child’s inhibitory control	−0.25	0.01	
**Household income (Canadian dollars)**			
<60 K/year (ref)	–	–	
≥60 K/year	0.26	0.12	
**Educational attainment**			
Sec./College (ref)	–	–	
University	0.02	0.63	
**Child’s sex**			
Boys (ref)	–	–	
Girls	−0.08	0.38	

The analysis was done on pooled imputed data.

### Clinical significance

In our regression results, each hour of daily screen time contributed to a 14% of standard deviation increase in inattention scores, as well as parenting stress contributed to a 24% of standard deviation increase in this outcome (Table 3). Despite its small size, this association is likely more clinically meaningful for heavy screen media exposure. Heavy screen use, characterized by a use of 4 h or more of screen media daily, would therefore result in a standard deviation increase of 14% multiplied by 4 h. As such, heavy media using children could experience a 56% of a standard deviation increase in their inattention symptoms.

## Discussion

The purpose of our study was to investigate associations between preschooler screen time, parenting stress, and later inattention symptoms during the COVID-19 pandemic. Our results showed that children exposed to more screen time during the pandemic at age 3.5 exhibited more inattention symptoms at age 4.5. Children whose parents had higher levels of parenting stress during the pandemic also experienced more symptoms of inattention 1 year later.

The current study provided support that preschooler screen time during the pandemic can have a negative impact on child attention development. Child screen use could displace time for attention building activities such as parent–child interaction, joint play, and outdoor play (Neuman, 1988; Christakis, 2009; Radesky J. et al., 2015). Indeed, a recent longitudinal study found that screen use decreased offline activities such as reading books, pretend play, and parent–child interaction (McArthur et al., 2021b). During screen-based activities, young children engage in fewer verbal and non-verbal interactions with parents which could also contribute to less optimal development in self-regulatory and attentional skills (Kirkorian et al., 2009; Radesky J.S. et al., 2015). More specifically, parent–child interactions play a central role in helping children internalise their self-regulation of attention during early childhood (Gartstein et al., 2008; Spruijt et al., 2020).

Preschoolers are likely to be more vulnerable to the screen exposure and the resulting displacement of developmentally enriching activities due to their increased brain plasticity during this time (Dumuid, 2020; Santos et al., 2022). Research with preschoolers and older children has linked screen time to decreased brain connectivity (Horowitz-Kraus and Hutton, 2018), lower microstructural integrity of brain white matter tracts (Hutton et al., 2020), and lower grey matter integrity (Paulus et al., 2019). There is also evidence that preschool screen time is associated with patterns of brain activation consistent with those observed in attention disorders (Zivan et al., 2019).

In light of the sensory overstimulation hypothesis, excessive and intense auditory and visual stimulation might condition the developing brain expect an intensity of inputs that reality cannot provide, forecasting later inattention problems (Christakis et al., 2018). Common features of media directed at children include, for example, changes in light, frequent camera cuts and quick pacing. As such children exposed to more screen time are also likely to have been exposed to contents with these characteristics. All these elements presents in new media devices can undermine sustained attention (Christakis et al., 2018).

In this sense, our results are consistent with the sensory overstimulation hypothesis (Christakis et al., 2018) and with previous research that showed positive associations between media use and reduced attentional skills. For example, screen time contributed to reduced effortful control in preschoolers (Fitzpatrick et al., 2022). Increased screen time contributed to worse inattention problems in a cohort of preschool children (Tamana et al., 2019). Multiple exposure to screen media, such as screen time and background use, is also related to decreased subsequent focused attention abilities in children (Gueron-Sela and Gordon-Hacker, 2020). As such, the present research provides further empirical support that screen time can undermine the development of attentional skills during preschool years.

In our study, parenting stress during lockdown was also associated with inattention symptoms in preschoolers one year later. Parents reported higher levels of stress during the pandemic (Spinelli et al., 2020; Malhi et al., 2021; Riter et al., 2021). According to an Italian study, parent distress contributed to higher inattention/hyperactivity in children aged between 3 and 13 (Marchetti et al., 2020). More highly stressed parents presented a reduction in the ability of the parent to enjoy and appreciate the parent–child relational experience, which in turn, have a negative impact on the child’s well-being (Spinelli et al., 2020). Parental stress can also decrease parent–child interactions (Chung et al., 2022). This could contribute to parents using and allowing more screens at home (Hartshorne et al., 2021).

### Practical implications

Early inattention symptoms are related to later impairment and more academic difficulties (Duncan et al., 2007; O’Neill et al., 2017; Landis et al., 2021). As such, it remains important to identify modifiable risk factors that contribute to their development. Preventive interventions implemented during the preschool years are more likely to be effective than those that take place in later childhood (Lakes et al., 2011). Interventions such as the *Incredible Years* (Jones et al., 2007) and *Behavioral Parent Training* (Hornstra et al., 2021), have been shown to improve child attention by improving the quality of parent–child interactions (Murray, 2010). Our findings suggest that interventions could benefit from coaching parents on how to establish healthy screen media use routines with children. Some recommendations, such as establishing routines for screen use with time limits, preferring media-free alternatives to shared activities, taking breaks during the media exposure to interact with the child (e.g., asking questions about the content watched) and adopting screen-time free moments can be important to reduce the potential harmful effects of media (Vanderloo et al., 2020). Furthermore, interventions could also address parenting stress, since overwhelmed parents may have more difficulty establishing and following a family media plan.

The COVID-19 pandemic disrupted many family routines, including screen use habits. For this reason, future research should seek to track the medium and long-term effects of child screen time during this time on later cognitive and behavioral outcomes. In particular, it remains important to investigate association between child screen time during the pandemic and later academic adjustment and achievement upon entering school. Last, future studies may help clarify the mechanisms by which child screen time contributes to increases in inattention symptoms.

### Strengths and limitations

This study is not without limitations. First, our abridged inattention measure was based only on three items and does not allow us to detect clinically significant levels of inattention. However, prior studies have shown that our measure is sensitive enough to forecast later behavioral and academic difficulties in children (Pagani et al., 2010). Second, our measures of child screen time, parenting stress, and inattention symptoms were parent-reported, which could have led to shared measurement bias, as well recall or social desirability bias. For example, parents reporting higher stress could allow longer child screen time and may also perceive their child as having more inattention symptoms. Third, we did not consider the content child uses on screen media. As previously discussed, some contents may be overstimulating for child attentional systems. In addition, the context of child screen use (i.e., using alone without adult supervision) could moderate associations between child screen time and later attention. Fourth, our findings are based on a correlational design which does not allow us to establish causal associations. Furthermore, our outcome was evaluated only at follow-up, which does not allow us to examine the possibility of reverse causation or bi-directional associations. Nevertheless, we did control for child inhibitory control at baseline, which reduces the possibility that children with more difficulty regulating their attention were exposed to higher amounts of screen time. Finally, this was based on a convenience sample that was relatively homogeneous and low risk, in terms of its socio-demographic characteristics. This could limit the generalizability of our findings to more vulnerable populations of preschooler.

On the other hand, this study has several strengths. The current study is one of the first to prospectively address the association between preschooler screen time and inattention symptoms during the pandemic. We are also one of the first studies to examine parenting stress during COVID-19 lockdown, a highly stressful context, and its association with later child inattention. Our study is enhanced by using a more exhaustive measure of child screen time, including multiple activities besides tv viewing, and screen devices, such as smartphone and tablets.

## Conclusion

Since attention is a crucial component for children development, behavior and academic outcomes, our study reinforces the importance of adopting healthy media habits and providing social support to parents of young children. Our results were observed above and beyond family characteristics which suggests that children may be vulnerable to the negative impacts of screens regardless of their sociodemographic background. The pandemic has increased screen use and parenting stress around the world. The association presented in this study can contribute to the development of evidence-based practices and recommendations for parents.

## Data availability statement

The data presented in this article are not readily available. As per the participant consent form, data are only available to the research team. Requests to access the data should be directed to caroline. fitzpatrick@usherbrooke.ca.

## Ethics statement

The studies involving human participants were reviewed and approved by Comité d’Étique, Université Sainte-Anne; Comité d’Étique, Université de Sherbrooke. Written informed consent to participate in this study was provided by the participant’s legal guardian/next of kin.

## Author contributions

ML and CF designed the study. ML conducted the analyses and drafted most of the manuscript. GG-C, EC, GF, and CF provided critical theoretical feedback on the entire manuscript. All authors contributed to the article and approved the submitted version.

## Funding

This research was supported by an establishment grant from Research Nova Scotia (PSO-EST-2019-2061), an insight development grant from the Social Sciences and Humanities Research Council (430-2018-00486), an operating grant from the Canadian Institutes of Health Research (474993), and the Canada Research Chairs program (CRC-2021-00009).

## Conflict of interest

The authors declare that the research was conducted in the absence of any commercial or financial relationships that could be construed as a potential conflict of interest.

## Publisher’s note

All claims expressed in this article are solely those of the authors and do not necessarily represent those of their affiliated organizations, or those of the publisher, the editors and the reviewers. Any product that may be evaluated in this article, or claim that may be made by its manufacturer, is not guaranteed or endorsed by the publisher.
